# The value of ATAD3A as a potential biomarker for bladder cancer

**DOI:** 10.1002/cam4.6759

**Published:** 2023-11-28

**Authors:** Zhenghong Liu, Li Sun, Bin Zheng, Heng Wang, Xiaowen Qin, Pu Zhang, Qijun Wo, Haichang Li, Yixuan Mou, Dahong Zhang, Shuai Wang

**Affiliations:** ^1^ Urology & Nephrology Center, Department of Urology Zhejiang Provincial People's Hospital, Affiliated People's Hospital, Hangzhou Medical College Hangzhou Zhejiang China

**Keywords:** ATAD3A, biomarker, bladder cancer, prognosis, survival

## Abstract

**Background:**

Bladder cancer (BCa) is a highly malignant tumor, and if left untreated, it can develop severe hematuria and tumor metastasis, thereby endangering the patient's life. The purpose of this paper was to detect the expression of ATAD3A in BCa and research the relationship between ATAD3A and pathological features of bladder cancer and the prognosis of patients.

**Methods:**

First, the expression of ATAD3A in BCa and normal bladder tissues was analyzed based on the UALCAN and Oncomine public databases. Second, 491 cases of surgically resected bladder cancer specimens and 110 cases of normal adjacent tissues were immunohistochemically stained. The expression of ATAD3A was quantified, and the value and prognosis of ATAD3A as a biomarker of BCa were evaluated.

**Results:**

The expression of ATAD3A in bladder cancer tissues was higher than that in normal bladder mucosa. High expression of ATAD3A was correlated with patient age, tumor size, number of tumors, distant metastasis, lymph node metastasis, lymphovascular invasion, and TNM stage (*p* < 0.05). Overexpression of ATAD3A is closely related to cancer patient survival. The mean survival time of bladder cancer patients with high ATAD3A expression was shorter than those with low ATAD3A levels. According to the relative comparing result, the high ATAD3A expression herald reduced overall survival in BCa patients.

**Conclusions:**

The abnormal overexpression of ATAD3A may be related to the initiation and progress of bladder cancer. The upregulation of ATAD3A can be used as an effective indicator to diagnose bladder cancer and predict tumor progression. Furthermore, the combination of information from public databases and the results of clinical sample analysis can help us better understand the mechanism of action of molecular oncogenes in bladder cancer.

## 1 INTRODUCTION

Bladder cancer (BCa) is the ninth most common malignancy worldwide.^1^ Every year approximately 573,000 new cases are diagnosed, and more than 213,000 patients die of BCa globally.^2^ The BCa incidence increases with age, and it is strongly associated with male gender, with men four times more frequently affected than women.^3^ The main risk factors for BC are tobacco smoking and occupational exposure.^4^ In terms of pathological types, urothelial carcinoma is dominant in North America and Western Europe, and squamous cell carcinoma is dominant in Africa.^1^

In the early stage of onset, patients with bladder urothelial carcinoma have fewer symptoms, while in the late stage, large‐scale metastasis occurs, which greatly increases the risk of death.^5^, ^6^ The progression of BCa is a complex pathological process with many influencing factors and is difficult to treat. Relevant statistical results showed that the proportion of patients with muscle‐invasive transitional cell carcinoma with distant metastasis after cystectomy reached 50%.^7^, ^8^ The diagnosis of such patients is mainly based on cystoscopy or tumor resection combined with pathological examination, but both methods have obvious limitations. Studies have found that some nonmuscle BCa (NMIBC) is highly invasive, so it is necessary to apply local chemotherapy mode during treatment.^9^ About 80% of BCa patients have reduced symptoms after intravesical chemotherapy, but the specificity of this method is not high.^10^

At present, the common methods in the field of BCa treatment include surgery, chemotherapy, radiotherapy, and combined immunotherapy, which have relative advantages and disadvantages and scope of application. According to the size, grade, and several tumors, different treatment schemes will be adopted. Radical total cystotomy is the standard treatment for muscle‐invasive bladder cancer. Nevertheless, the operation is difficult, time‐consuming, and complicated and has a high‐fatality rate, which puts forward high requirements for the surgeon and nursing. The surgical fatality rate has decreased considerably, but the incidence of postsurgical complications is still high. At present, in the research field of this disease, the hot topic is to identify new markers and targets of early BCa, which is important for its diagnosis and treatment.^11^

The AAA domain contained protein 3A (ATAD3A) of the ATPase family is a special membrane‐anchored protein, which has multiple biological activities, such as affecting mitochondria, and cholesterol metabolic processes.^12^ This membrane protein can also promote protein translation and cell proliferation. Knocking out ATAD3A leads to disordered protein processing, leading to endoplasmic reticulum (ER) stress. For eukaryotic cells, it is necessary to transport the translated and modified proteins and phospholipids to specific subcells, which is important for cell function. Relevant studies have found that in the organelles of animal cells, the DNA genome is mainly contained in mitochondria, but these DNA‐encoded proteins are mainly encoded by nuclear genes.^13^ ATAD3A is a protein that spans the mitochondrial membrane, with the C‐terminus facing the matrix and the N‐terminus outward.^14^

In response to ER stress induced by chemotherapy, ATAD3A interacts with the GRP78 protein to mitigate the effects of ER stress on cancer cell survival.^15^ Reduced ER stress results in reduced surface exposure to calreticulin, a promoter of immunogenic cell death. Relevant studies have found that silencing ATAD3A can promote cell death, increase T lymphocyte infiltration, and improve the level of cancer cell apoptosis.^15^ In cells, ATAD3A plays an important role in the maintenance of mitochondrial structure and function and is closely related to cell growth, mitochondrial protein synthesis, and the genetic process. Several studies have demonstrated that ATAD3A binds to DNA D‐loops, contributes to nucleoid stability, and is involved in mitochondrial‐mediated antiviral innate immunity.^16^ Structurally, it spans the outer membrane and inner membrane of mitochondria and can regulate the interaction of the two in a specific form.^17^ Mechanistically, ATAD3A interacts with mitochondrial channel components Tom40 and Tim23 and acts as a bridging factor to promote the proper transport and processing of autophagy protein Pink1. ATAD3A depletion leads to Pink1 accumulation and activation of mitochondrial fission. Jin et al. demonstrated that ATAD3A inhibits PinK1‐dependent autophagy and therefore plays a critical role in hematopoietic homeostasis.^18^ ATAD3A mutations cause various phenotypes and are closely related to mitochondrial diseases in infants.^19^ Although ATAD3A mutations are rarely found in cancer patients, ATAD3A is still associated with certain types of cancer usually Herald poor patient outcomes. According to the experimental result, the expression level of ATAD3A in cancer cells was significantly increased, and the corresponding signal pathway was activated to promote the proliferation of tumor cells.

In recent years, a large number of experimental studies have found that the mitochondrial function and biological behavior of cancer cells are mainly related to ATAD3A, thus playing a bridge role. This membrane protein also plays an important role in research into new cancer therapies that inhibit tumor proliferation.^20^ ATAD3A is also essential for maintaining mitochondrial structure, so many diseases are associated with mutations and deletions of the ATAD3A gene. Relevant genetic studies have found that both dominant and recessive mechanisms exist for changes in the ATAD3A gene, and the phenotypes caused by them are different, indicating that the correlation between mutations and diseases is very complex. Harel‐Yoon syndrome is a neurodevelopmental disorder. The main symptoms in such patients are trunk hypotonia and impaired motor function. One of the main causes of this syndrome is mutations in the ATAD3A gene.^21^ Recurring neo‐dominant missense mutations in ATAD3A adversely affect the patient's physical development while leading to decreased muscle tone, optic atrophy, and a greatly increased likelihood of cardiovascular disease.^21^ Moreover, according to relative research, in the relevant literature, the high‐level expression of this gene is associated with lung adenocarcinoma, and prostate cancer.^22^ However, its expression pattern and role in BCa remain unclear.

Given the important physiological role of ATAD3A in tumorigenesis, we proposed the hypothesis that the expression level of ATAD3A could be used to predict tumorigenesis and survival. In this paper, we investigated the role and possible mechanisms of ATAD3A in bladder cancer development, assessed ATAD3A activation and expression, and compared the expression of this gene in normal bladder tissue and BCa cells. We aimed to investigate whether ATAD3A could be a valuable biomarker for the diagnosis, prognosis, and treatment of BCa.

## 2 METHODS

### 2.1 Patients and tissue samples

In this paper, tissue specimens from 491 bladder cancer patients admitted in Zhejiang Provincial People's Hospital from February 1998 to December 2011 were collected. 294 cases of bladder cancer tissues were collected by transurethral resection of bladder tumor (TURBt) and 197 cases were collected by radical cystectomy (RC). The age of their range is from 35 to 79 years old. The sample included 102 patients with single tumors and 389 with multiple tumors. In addition, according to the pathological grading standard of the WHO in 2004, there were 38 cases of low‐grade urothelial carcinoma and 453 cases of high‐grade urothelial carcinoma. There were 67 cases of nonmuscle invasive BCa and 424 cases of muscle‐invasive BCa. All research objects were followed up for 5 years after surgery, from surgery to death. The main cause of death of the patients was tumor metastasis and recurrence.

The grading of bladder cancer is mainly based on the nuclear morphology of tumor cells, as well as differences in nuclear division pattern, and tumor cells are classified into three grades. Where Grade I is low grade also called well differentiated, Grade II is intermediate grade that is, moderately differentiated and Grade III is high grade that is, poorly differentiated or undifferentiated. The role of grading is to assess the malignancy of tumor cells and the sensitivity of treatment as well as to determine prognosis, specifically, bladder cancer Grade I tumors with well‐differentiated cells, low grade of malignancy, sensitive to treatment, and good prognosis. Grade III was just the opposite, and Grade II was between Grades I and III. Clinicians often combine the grading of bladder cancer with the staging of bladder cancer for analysis, which is used to guide clinical treatment as well as to judge the prognosis of tumors.

We collected 110 control samples from the tumor margin. All specimens were fixed and embedded in paraffin. The tissue microarray, in the paraffin block (tissue array block), includes the most invasive core tissue biopsy of the tissue block (diameter 2 mm). This study was approved by the Ethics Committee of Zhejiang Provincial People's Hospital (IRB: QT2023031), and all patients signed informed consent forms.

### 2.2 The expression of ATAD3A in BCa was evaluated using bioinformatics databases

The differential expression of ATAD3A mRNA was analyzed by using online microarray databases such as Oncomine and UALCAN. The specific parameters searched in these databases included sample type, individual cancer stages, patient race, sex, weight, age, smoking habit, tumor histology, molecular subtype, nodal metastasis status, TP53 mutation status, and cancer type. The prognostic value of ATAD3A expression was further analyzed by using the UALCAN database. The median survival time was used for survival analysis.

### 2.3 Immunohistochemistry and evaluation of ATAD3A protein expression

The expression of ATAD3A was detected by immunohistochemistry. During the experiment, 4 mm sections were cut from each sample tissue block and fixed on the slide. And then put them into an oven at 60°C for 2 h. After that, the sections were taken out and dewaxed in xylene, followed by hydration based on gradient ethanol solution and microwave heating in 10 mm citric acid for 10 min. Put the slices treated above into 3% hydrogen peroxide for 10 min to inactivate them. And 1% bovine serum albumin was added. After that, the sections were incubated with anti‐ATAD3A mouse monoclonal antibody (Santa Avenue, CA, dilution range 1:50–1:500) for 16 h at 4°C. The sections were taken out and fully washed, and then treated with secondary antibody (envision, dilution range 1:200–1:500). The sections were refixed with hematoxylin and stored as required after dehydration.

The immunostaining score was distributed independently by two individuals who did not have access to the pathological and clinical data. The score was based on the number of stained cells. If there was a difference between the observers, a consensus score was selected for evaluation. The staining intensity was graded based on criteria: to no staining, light yellow, brownish yellow, and brownish‐yellow staining correspond to 0, 1, and 2, 3 points, respectively. Less than 5% of the stained cells were assigned 0 points, 6%–26% of the stained cells were assigned 1 point, 26%–50% of the stained cells were assigned 2 points, and 50% or more cells stained were assigned 3 points. Based on multiplying positive cells score by the score of the staining intensity, the final score was got: 0–4 was considered a low level of ATAD3A expression, and 5–9 was considered a high level of ATAD3A expression.

### 2.4 Statistical analysis

The SPSS V.26.0 software was used for statistical analysis. The relationship between ATAD3A level and clinicopathological factors was checked with *χ* ^2^ tests. The overall survival curve was generated by the Kaplan–Meier method. To assess the prognostic value of ATAD3A expression, the Cox regression model was used to analyze survival impressive factors. In all tests, *p* < 0.05 was considered statistically significant.

## 3 RESULTS

### 3.1 Expression analysis of ATAD3A based on relative database

**FIGURE 1:**
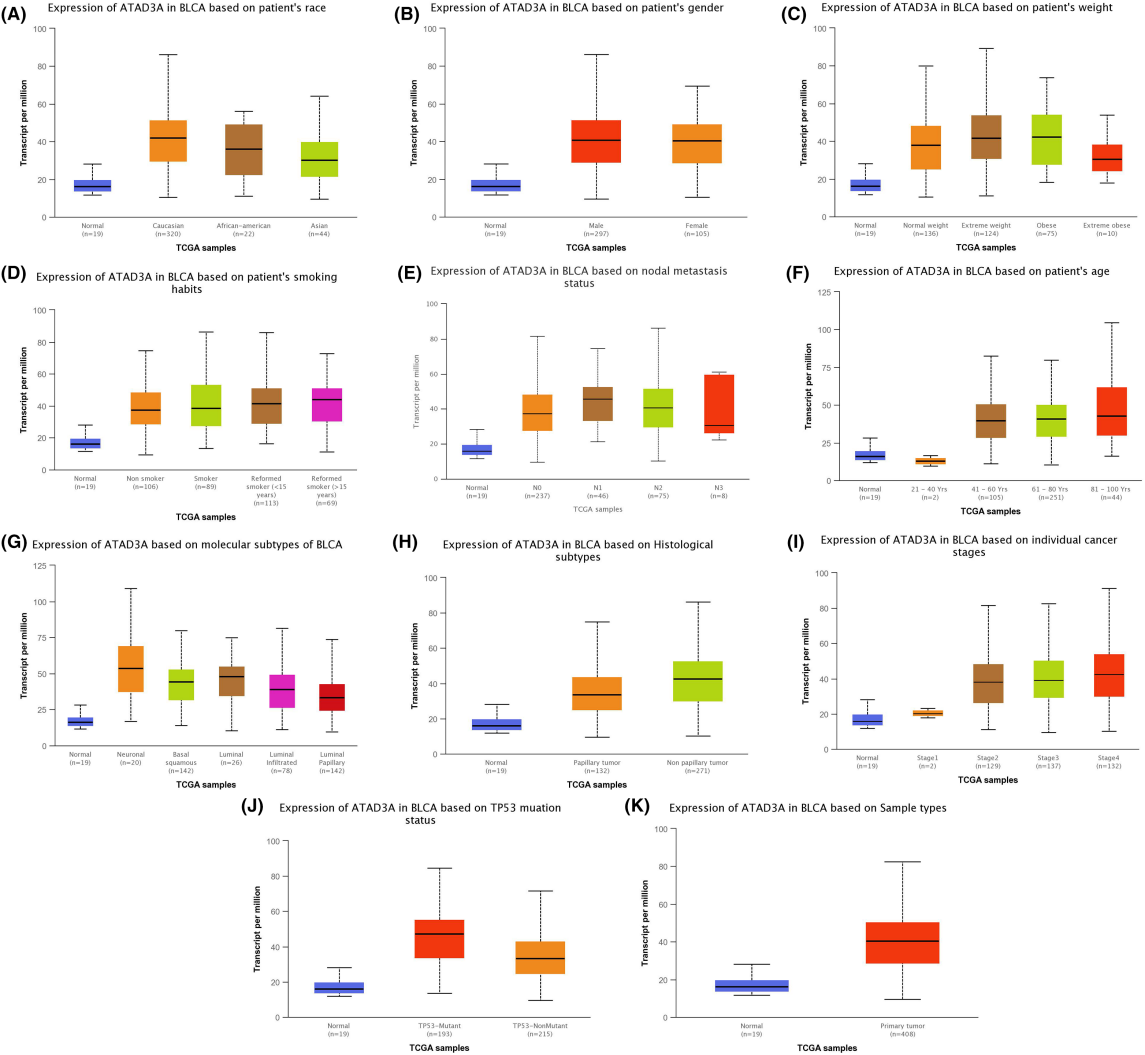
(A–K) Expression of ATAD3A mRNA according to patient's race, gender, weight, smoking habits, nodal metastasis status, age, molecular subtypes, histological subtypes, individual cancer stages, tumor statutes, and sample types based on the Ualcan database.

To evaluate the expression of ATAD3A mRNA, we searched the UALCAN database. Analysis of the UALCAN data indicated that there have no obvious differences in the ATAD3A level among different patient races, sexes, body weights, smoking statuses, or lymph node metastases, as shown in Figure 1A–E. Moreover, further analysis of the UALCAN database documented that the median expression in normal samples (*n* = 19) was 16.102, and the median expression was 12.962 from 21 to 40 years old (*n* = 2), 39.789 from 41 to 60 years old (*n* = 105), 40.731 from 61 to 80 years old (*n* = 251), and 42.802 from 81 to 100 years old (*n* = 44). Among the molecular subtypes, the median expression in neuronal (*n* = 20) was 53.622, basal squamous (*n* = 142) was 44.3, luminal (*n* = 26) was 47.996, luminal infiltrated (*n* = 78) was 38.967 and luminal papillary (*n* = 142) was 33.422. Furthermore, the median expression in papillary tumors was 33.432, and that of nonpapillary tumors was 42.617. The median expression of ATAD3A in Grade 1 cancer (*n* = 2) was 20.402, in Grade 2 (*n* = 129) was 38.115, in Grade 3 (*n* = 137) was 39.234, and in Grade 4 (*n* = 132) was 42.653. Remarkably, the median expression in TP53‐Mutant (*n* = 193) was 47.207, and TP53‐NonMutant (*n* = 215) was 33.158. When sample types were considered, the median expression in normal samples (*n* = 19) was 16.102 and in primary tumors (*n* = 408) was 40.301.

**FIGURE 2:**
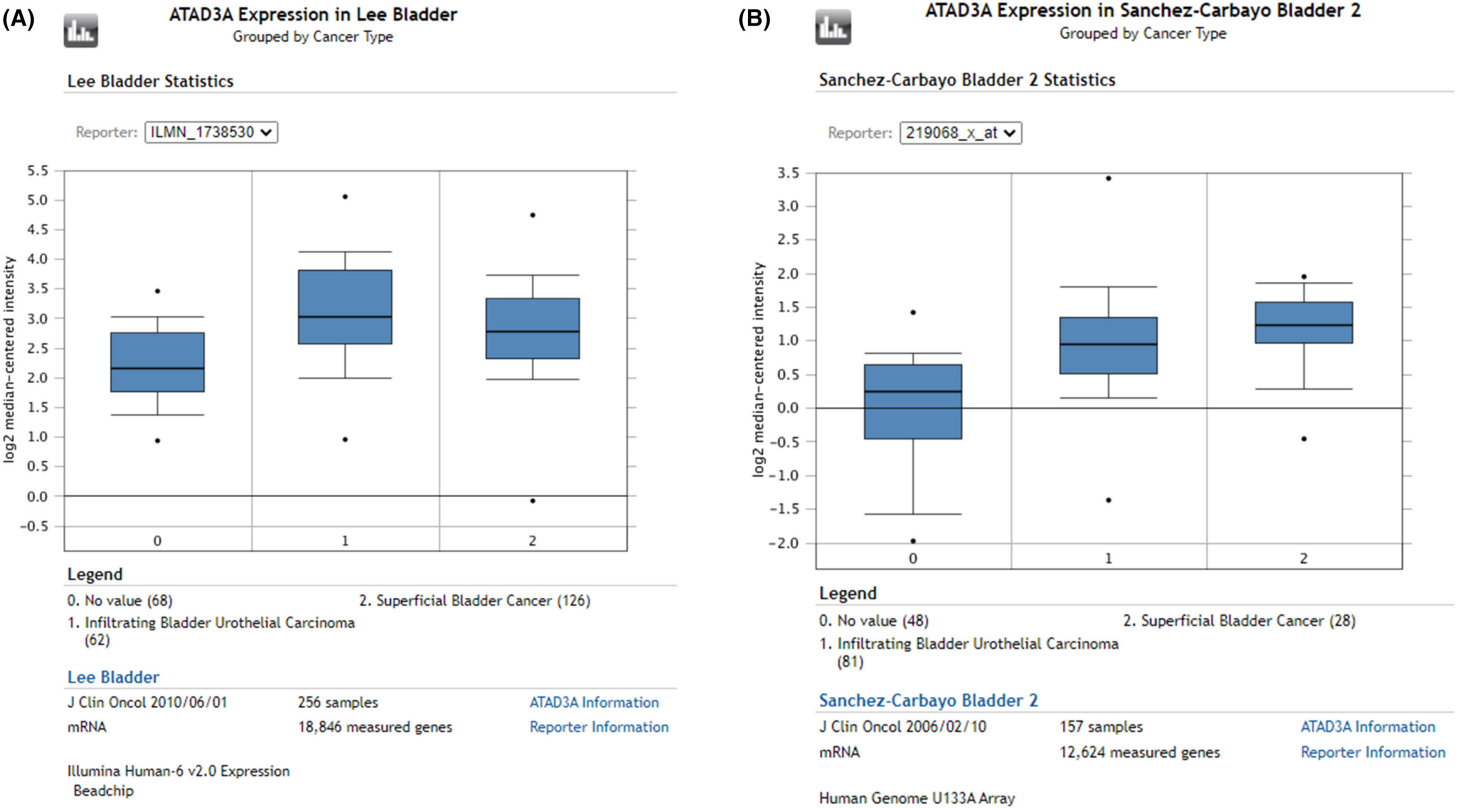
(A‐B) The expression of ATAD3A from different groups of cancer types on Oncomine databases.

In summary, the expression of ATAD3A mRNA in normal bladder tissue in the age group over 40 was higher than that in the younger age group, as shown in Figure 1F, and ATAD3A mRNA levels were significantly higher in any molecular subtype, histological subtype, individual cancer stage, TP53 mutation status, and sample type than in normal bladder tissue, as shown in Figure 1G–K. In parallel, according to the Oncomine database, ATAD3A expression in superficial bladder cancer and infiltrating bladder urothelial carcinoma was higher, as shown in Figure 2A or Figure 2B.

**FIGURE 3:**
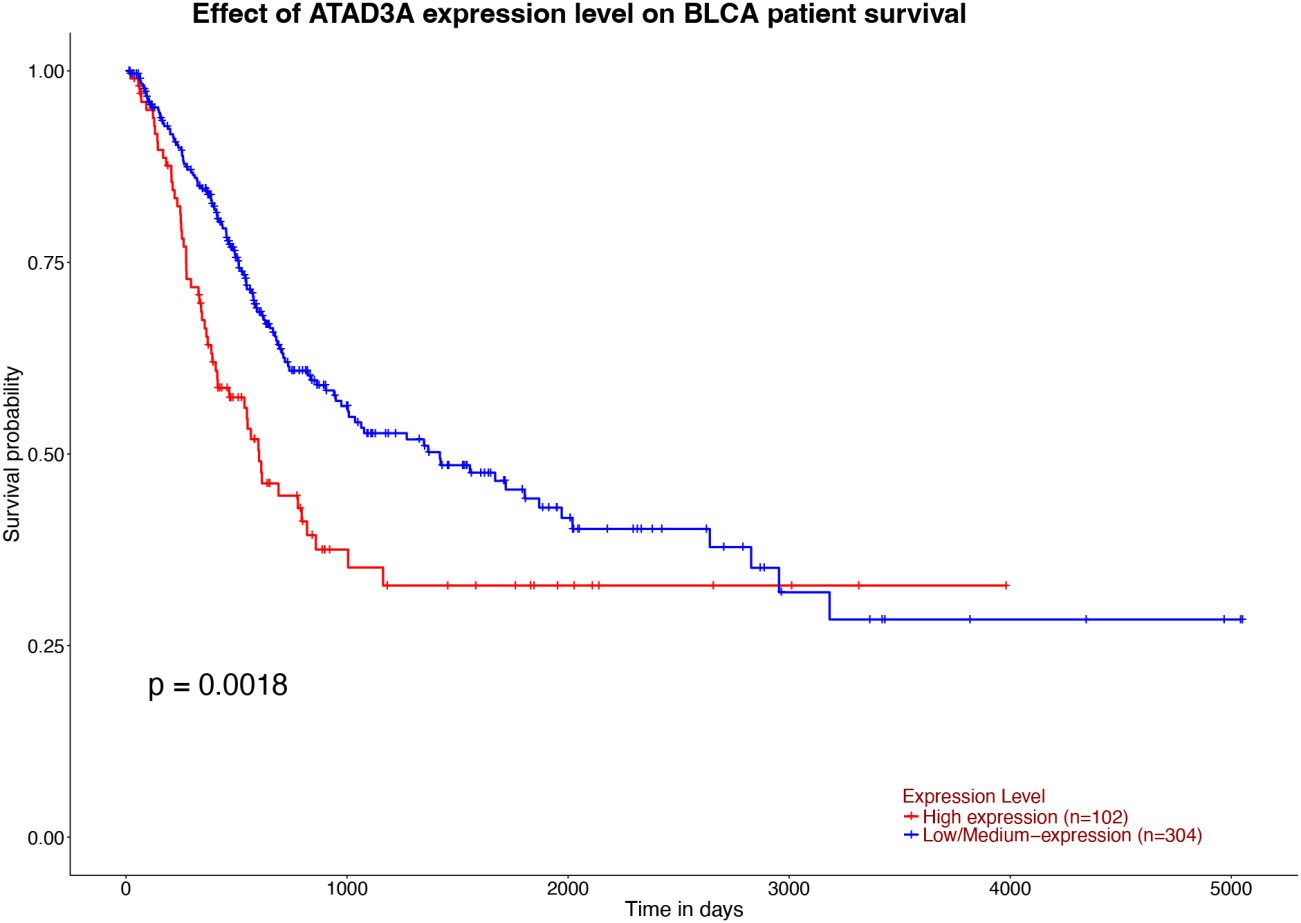
The prognostic value of ATAD3A levels in BCa patients. Survival curves were plotted based on the Ualcan databases.

The UALCAN database was applied to evaluate the correlation between of ATAD3A level and the patient prognosis. As shown in in Figure 3, UALCAN demonstrated that high ATAD3A mRNA expression levels usually herald worse clinical prognosis.

### 3.2 Overexpression of ATAD3A in BCa tissues

**FIGURE 4:**
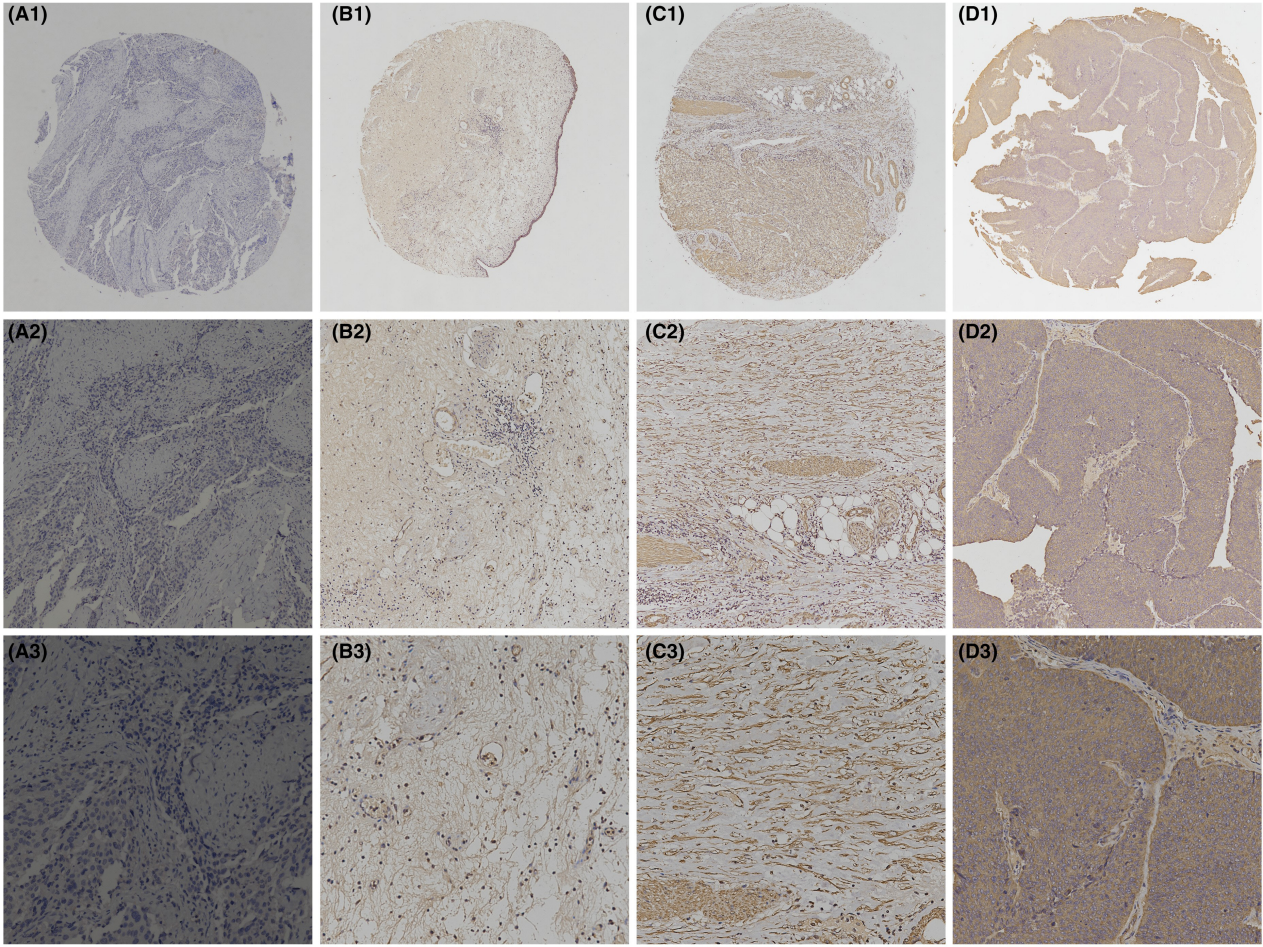
Immunohistochemical staining for ATAD3A in BCa tissues and normal tissue adjacent to the tumor. (A1–A3) Negative control of ATAD3A in BCa (PBS was used instead of primary antibody). (B1–B3) ATAD3A showed low expression in normal tissue adjacent to the BCa. (C1–C3) Moderate expression of ATAD3A in BCa tissue. (D1–D3) High expression of ATAD3A in BCa tissue. Original magnification: A1–D1, ×40; A2–D2, ×200; A3–D3, ×400.

Immunohistochemistry was used to detect the ATAD3A protein level in cytoplasm of bladder cancer and nontumor tissues. According to the result of Figure 4, ATAD3A was mainly expressed in the cytoplasm of bladder cancer cells. In 110 bladder mucosa control specimens, ATAD3A protein was detected in nine cases (8.2%), but its level was always low. For 491 tumor tissue, ATAD3A was detected in 280 cases (57.0%), and higher than that in the control group (*p* < 0.01), as shown in Table 1. Figure 4 shows the score chart of ATAD3A immunostaining in the analytical tissue.

**TABLE 1:** Expression of ATAD3A in BCa and non‐carcinomatous bladder tissues.

Samples	ATAD3A expression			
	Number	Negative	Positive	*p*
BCa	491	211	280	<0.01
Non‐carcinomatous bladder tissues	110	101	9	

### 3.3 Relationship between ATAD3A protein expression and prognosis in BCa

The correlation analysis results of ATAD3A level and clinical factors are listed in Table 2. Higher expression of ATAD3A related to age (*p* = 0.029), tumor size (*p* = 0.001), tumor number (*p* = 0.039), lymph node metastasis (*p* = 0.001), distant metastasis (*p* = 0.039), lymphatic invasion (*p* = 0.040), and TNM stage (*p* = 0.017). However, there was no obvious significant correlation with sex (*p* = 0.347), depth of invasion (*p* = 0.167), degree of differentiation (*p* = 0.123) or vascular invasion (*p* = 0.680).

**TABLE 2:** Relationship of ATAD3A expression with pathological parameters of BCa.

Clinical parameters		ATAD3A expression		
	Low	High	χ2	*P*
Gender			0.884	0.347
Male	189 (89.6%)	243 (86.8%)		
Female	22 (10.4%)	37 (13.2%)		
Age (years)			4.775	0.029
<60	92 (43.6%)	95 (33.9%)		
≥60	119 (56.4%)	185 (66.1%)		
Size			10.107	0.001
<3 cm	121 (57.3%)	120 (42.9%)		
≥3 cm	90(42.7%)	160 (57.1%)		
Number of tumor			4.243	0.039
Single	53 (25.1%)	49 (17.5%)		
Multiple	158 (74.9%)	231 (82.5%)		
Invasion depth			1.913	0.167
Ta–T1	34 (16.1%)	33 (11.8%)		
T2–T4	177 (83.9%)	247 (88.2%)		
Lymph node metastasis			10.593	0.001
No	174 (82.5%)	195 (69.6%)		
Yes	37 (17.5%)	85 (30.4%)		
Distant metastasis			4.255	0.039
No	200 (94.8%)	251 (89.6%)		
Yes	11 (5.2%)	29 (10.4%)		
Lymphovascular invasion			4.214	0.040
Negative	104 (49.3%)	112 (40.0%)		
Positive	107 (50.7%)	168 (60.0%)		
Degree of differentiation			4.189	0.123
Low‐grade non‐invasive	22 (10.4%)	16 (5.7%)		
High‐grade non‐invasive	57 (27.0%)	72 (25.7%)		
Invasive	132 (62.6)	192 (68.6%)		
Vascular invasion			0.170	0.680
No	169 (80.1%)	220 (78.6%)		
Yes	42 (19.9%)	60 (21.4%)		
TNM stage			10.190	0.017
I	63 (29.9%)	53 (18.9%)		
II	54 (25.6%)	66 (23.6%)		
III	64 (30.3%)	111 (39.6%)		
IV	30 (14.2%)	50 (17.6%)		

### 3.4 The significance of ATAD3A expression for BCa prognosis

**FIGURE 5:**
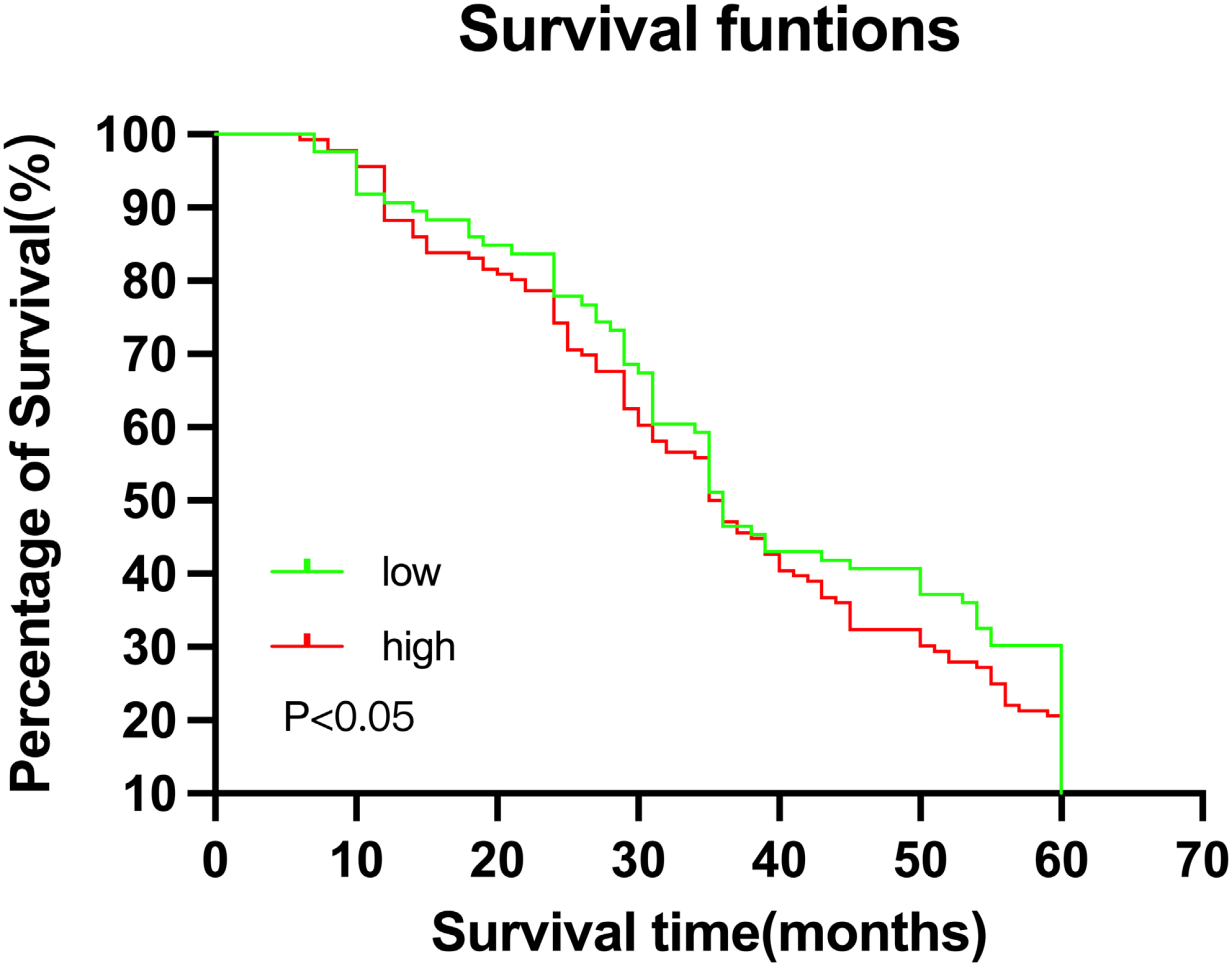
Kaplan–meier survival curve showed that patients with low ATAD3A expression had a better prognosis than those with higher ATAD3A expression (*p* < 0.05).

The median survival time of patients with low ATAD3A levels (50.664 ± 2.170 months) was obvious longer than that of patients with high ATAD3A levels (46.954 ± 2.091 months) (*p* < 0.05). In addition, Kaplan–Meier analysis results indicate that high ATAD3A level was related to shortened overall survival (*p* < 0.05), as shown in Figure 5. Univariate analysis was conducted on the important factors affecting survival. The survival time was correlated with age (*p* = 0.048), tumor size (*p* = 0.015), degree of differentiation (*p* = 0.044), distant metastasis (*p* < 0.01), vascular invasion (*p* < 0.01), invasion depth (*p* < 0.01), lymph node metastasis (*p* < 0.01), lymphatic invasion (*p* < 0.01) and ATAD3A expression (*p* < 0.01). These factors were included in the Cox hazard regression model and demonstrated that age, distant metastasis, vascular invasion, invasion depth and lymphovascular invasion were independent factors affecting the prognosis of bladder cancer patients, while tumor size, degree of differentiation, lymph node metastasis and ATAD3A expression were not independent factors affecting the outcome of bladder cancer patients, as shown in Table 3.

**TABLE 3:** Univariate and multivariate Cox regression survival analysis of clinicopathological parameters and ATAD3A expression in BCa patients.

Parameters	Univariate analysis			Multivariate analysis		
	HR	95% CI	P	HR	95% CI	P
Age	1.320	1.003–1.739	0.048	1.329	1.051–1.844	0.021
Gender	1.181	1.804–1.736	0.396	NA		
Tumor size	1.389	1.065–1.811	0.015	1.087	0.826–1.430	0.551
Number of tumors	1.387	0.998–1.927	0.051	NA		
Degree of differentiation	1.237	1.006–1.521	0.044	1.093	0.867–1.378	0.450
Metastasis	1.902	1.212–2.983	0.005	1.887	1.160–3.070	0.011
Vascular invasion	2.974	2.211–4.000	0.000	2.537	1.845–3.490	0.000
Invasion depth	2.450	1.530–3.923	0.000	2.060	1.248–3.401	0.005
Lymph node metastasis	1.650	1.221–2.229	0.001	1.141	0.823–1.583	0.428
Lymphovascular invasion	2.084	1.583–2.745	0.000	1.588	1.185–2.128	0.002
ATAD3A expression	1.524	1.162–1.999	0.002	1.260	0.951–1.670	0.108

Abbreviations: CI, confidence interval; HR, hazard ratio.

## 4 DISCUSSION

In this report, we demonstrated that high ATAD3A expression usually heralds a poor prognosis in bladder cancer patients, and ATAD3A level can be treated as an independent prognostic factor. Other factors that were associated with patient survival included age, metastasis, vascular invasion, invasion depth and lymphovascular invasion. The results showed that the average survival time of patients was longer when the expression of ATAD3A was low. ATAD3A can be used as an effective objective index in identifying patients with bladder cancer, which is helpful to judge the patient's condition through its expression level and provide support for optimizing the treatment plan.

BCa is a kind of malignant tumor in humans. Surgical treatment is the main treatment at present, but this operation is a high‐risk operation. The perioperative complication rate can reach 28% to 64%,^23^ and the perioperative mortality rate is 2.5% to 2.7%.^24^ At present, many targeted materials and drugs have emerged to treat BCa, such as nucleic acid aptamers, nucleic acid, and peptides. Therefore, the early screening of bladder cancer is particularly important. Many studies have shown that novel tumor markers can be used to diagnose bladder cancer, such as survivin, NMP‐22, and MMP‐28.^25^, ^26^, ^27^ However, thus far, there are no ideal tumor markers to replace cystoscopy and urine exudation cytology due to the limitations of the overall sensitivity. Bladder tumor markers have clinical significance, and they are essential for screening processes, early diagnosis, surveillance, staging, and prognostication.^28^ Thus, it is necessary to develop high‐efficiency and non‐invasive BCa biomarkers to provide support for its early diagnosis and promote prognosis analysis. This is also the focus of research in the field of urology.

The ATPase family, AAA domain containing 3A (ATAD3A; 66 kDa), has been found to play an important role in the maintenance of mitochondrial structure and function, and also provide support for signal transmission between endoplasmic reticulum (ER) and mitochondria.^29^ AAA domain proteins of the ATPase family are a large diverse NTPase superfamily. More than 53 members of this superfamily have been identified, which play various roles in the overall process of cell activities. Including protein degradation, cytochrome assembly, helicase activity, and gene expression.^30^ The examination of other relevant cancer tissues also found that the expression level of the ATAD3A gene was significantly increased, and some detected variants, which would have an impact on the physiological activities of cancer cells and the pathological process of cancer.

ATAD3A was originally identified as a tumor‐specific antigen.^31^, ^32^ It has been confirmed as one of the strongly overexpressed tumor antigens in head and neck cancer.^22^ Lang et al.^33^ highlighted the novel function of ATAD3A in regulating mitochondrial ERK1/2 activation that favors head and neck squamous cell carcinoma development. Combined targeting of ATAD3A and RAS signaling may potentiate anticancer activity for head and neck squamous cell carcinoma therapeutics. The high expression of ATAD3A can promote the proliferation of glioma cells and is closely related to the growth of prostate cancer cells. ATAD3A expression levels were significantly higher in prostate cancer tissues than in normal prostate cells or benign hypertrophy prostate epithelial cells.^34^ High expression of ATAD3A was detected in lung adenocarcinoma (LADC), and overexpression of ATAD3A was also detected in cervical cancer patients.^35^ Besides, the high ATAD3A level is related to the patient's disease status and recurrence level, tumor grade, and metastasis.^35^ Some scholars have found that the expression of ATAD3A is significantly correlated with the content of high‐risk human papillomavirus (hrHPV), disease stage, lymph node involvement, and patient survival rate in cervical cancer. Silencing hrHPV E6/E7 expression can reduce the expression of ATAD3A and the survival rate of cervical cancer cells, which may be related to p53 and pRB.^36^ Cancer patients with overexpression ATAD3A have a high recurrence rate. High expression of ATAD3A can also be found in LADC cell lines. Furthermore, LADC patients with overexpression of ATAD3A have a lower survival rate.^35^ Tumors with high expression of ATAD3A have a high metastasis phenomenon. ATAD3A increases breast cancer metastasis through metastasis promoter WASF3, and knockdown of ATAD3A can inhibit the migration of colon cancer cells and breast cancer cells.^37^ Silencing ATAD3A can also inhibit the growth of mammary tumors in mouse breast cancer models, and the tumors generated significantly smaller while ATAD3A knockdown. Some scholars established the mouse model of ATAD3A knockout breast cancer cells in the process of research and found that the metastasis rate of corresponding breast cancer cells was significantly reduced in the Lung Group.^37^ In cancer cell proliferation, the target of ATAD3A regulation is still unclear, and the corresponding regulatory mechanism still needs to be further studied. Rapamycin (TOR) can regulate nuclear mitochondrial protein expression and is closely related to the cell proliferation process.^14^ ATAD3A plays various regulatory roles in the development of multicellular organisms.

Related studies have found that ATAD3A silenced by RNA interference can trigger a variety of diseases of Caenorhabditis elegans, mainly including larval growth retardation and gonadal dysfunction.^38^ The research results of some scholars show that the target of rapamycin can regulate mitochondrial function based on ATAD3A in several model organisms of drosophila and affect its structure. Drosophila ATAD3A is regulated by the TOR signaling pathway and has obvious effects on the cell division process.^39^ After the gene deletion of dATAD3A and the interference of the mTOR inhibitor, the growth and development speed of Drosophila larvae significantly decreased, and the proliferation speed of adipocytes decreased.^40^ In addition, the ATAD3A/TOR axis can also affect the mitochondrial biological behavior during mouse embryonic development in varying degrees, which is crucial for its function. ATAD3A mutations can easily cause embryonic development defects and individual developmental delay.^41^ The research results of Chen et al.^42^ found that the expression level of PTEN‐induced kinase 1 was significantly downregulated in ATAD3A Ko Huh7 cells, which can be judged that it is also closely related to cell mitosis. Under free cholesterol overload, the deletion of ATAD3A will lead to the inhibition of the mitochondrial respiratory function of Huh7 cells, and the corresponding ATP production rate and level will decrease, and the activity of ATP synthase will decrease. According to the above research results, it can be inferred that the deletion of ATAD3A can promote the progress of alcoholic fatty liver, mainly through the accumulation of triglycerides in hepatocytes and abnormal mitochondrial metabolism. Zhao et al.^43^ found that the susceptibility of Alzheimer's disease (AD) disease may come from the disorder of lipid metabolism, and the corresponding mediator is ATAD3A, which mainly plays the role of a molecular switch. According to relevant studies, ATAD3A is also related to resistance to radiotherapy and chemotherapy. Mitochondrial phagocytosis is of great significance for mitochondrial quality control and is also closely related to the state stability of mitochondria.^44^ After being affected by relevant factors, mitochondria are self‐depolarized, and damaged organelles are recognized by autophagosomes and fuse with lysosomes to complete the degradation process.^45^ Mitochondrial phagocytosis is associated with many diseases, such as Parkinson's disease, diabetes mellitus, myocardial ischemia–reperfusion injury, ankylosing spondylitis, and cancer.^46^ The study of Wu et al.^47^ showed that the mitotic process was significantly inhibited in sorafenib‐resistant HCC cells. Increased expression of ATAD3A is closely related to sorafenib resistance. High expression of ATAD3A demonstrates stronger drug resistance and higher radiation resistance. ATAD3A can be recognized as an anti‐apoptotic factor in prostate cancer. Silencing ATAD3A in human prostate cancer cells (LNCaP) significantly reduces cisplatin resistance.^34^ Existing studies have fully proved that ATAD3A has a high tumor marker value, but the correlation between its expression level and clinicopathology of bladder cancer is not very clear, and the corresponding research reports are missing.

Using the UALCAN and Oncomine databases, we proved that the expression of ATAD3A in bladder cancer was higher than that in normal bladder tissue and higher in TP53 mutation than in TP53 nonmutation bearing tumors. Next, it is very needed to research the relation between ATAD3A and tumor progression, clinicopathological features, and overall survival. By immunohistochemistry, ATAD3A expression was detected in 491 cases of bladder cancer and 110 normal bladder tissues. The correlation analysis of the immunohistochemical score with clinicopathological and genetic characteristics showed that ATAD3A was highly expressed in BCa. Age (*p* < 0.05), tumor size (*p* < 0.05), tumor number (*p* < 0.05), lymph node metastasis (*p* < 0.05), distant metastasis (*p* < 0.05), lymphatic invasion (*p* < 0.05), and TNM stage (*p* < 0.05) were also correlated. This research result has given strong evidence that ATAD3A contributes to tumor development and spread.

Furthermore, to evaluate the prognostic value of ATAD3A in bladder cancer, we performed a Kaplan–Meier analysis of data from public sources and found that the mean survival time of patients with high ATAD3A expression was significantly shorter than that of patients with low ATAD3A expression. These results suggest that ATAD3A expression can be an effective indicator for identifying high‐risk populations and predicting the outcome of bladder cancer patients.

On the other hand, we should acknowledge that this study has some limitations. In our analysis, the number of samples in public database appears to be insufficient and may not be representative of the entire BCa patient population. Other pitfalls include the subjective nature of scoring based on immunohistochemical scoring and the limited number of clinical samples. Moreover, our results may be affected by variable overlap. Nonetheless, we believe that the sample size and number of clinical cases in the database will continue to expand, allowing our conclusions to be fully validated.

## 5 CONCLUSIONS

In conclusion, by combining public database information on tumor gene expression with results from clinical samples, we have improved our understanding of the role of molecular oncogenes in BCa. Furthermore, this study assessed ATAD3A expression in BCa patients and its association with overall survival. ATAD3A can be used to predict survival in BCa patients. Therefore, ATAD3A can be used as a molecular marker for the diagnosis, treatment, and prognosis of BCa, and is beneficial for the treatment of these patients. In the future, we need to explore various regulatory functions and mechanisms of the malignant behavior of bladder urothelial carcinoma and provide new approaches for the treatment of BCa.

## AUTHOR CONTRIBUTIONS

**Zhenghong Liu:** Writing – original draft (equal). **Xiaowen Qin:** Data curation (equal). **Wentao Xu:** Data curation (equal). **Heng Wang:** Conceptualization (equal). **Li Sun:** Formal analysis (equal). **Pu Zhang:** Investigation (equal). **Bin Zheng:** Supervision (equal). **Haichang Li:** Resources (equal). **Yixuan Mou:** Visualization (equal). **Dahong Zhang:** Writing – review and editing (equal). **Shuai Wang:** Writing – review and editing (equal).

## FUNDING INFORMATION

This study was funded by the Medical Technology Research Plan of Zhejiang Province (2022497314), Medical Technology Research Plan of Zhejiang Province (2021421701), and the Natural Science Foundation of Zhejiang Province (LQ21H160041).

## CONFLICT OF INTEREST STATEMENT

The authors declare no conflict of interest.

## ETHICS STATEMENT

This study was approved by the Ethics Committee of Zhejiang Provincial People's Hospital (IRB: QT2023031), and all patients signed informed consent forms.

## Data Availability

All data generated or analyzed during this study are included in this manuscript.
